# The role of cardiac MR in identifying annulus paradoxus, a specific marker for constrictive pericarditis

**DOI:** 10.1186/1532-429X-17-S1-P362

**Published:** 2015-02-03

**Authors:** Kashif Kalam, Werner Harmse, Naeem Merchant, Winnie Fu

**Affiliations:** 1 The Stephenson cardiovascular MR centre, Libin cardiovascular institute, Calgary, AB, Canada; 2 Diagnostic imaging, University of Calgary, Calgary, AB, Canada

## Background

Distinguishing constrictive pericarditis from restrictive cardiomyopathy can be challanging. In normal subjects and many cardiac pathologies, the lateral mitral annulus compared to medial demonstrates more longitudinal excursion. This is due to the presence of more longitudinal fibres and lubrication offered by pericardium. Annulus paradoxus is a term used to define reversal of this phenomenon due to tethering of the pericardium and has been extensively reported as a specific marker of constrictive pericarditis in echocardiography by using tissue doppler imaging (TDI). TDI is a pulse wave doppler technique and has limitations due to aliasing and angle of interrrogation. We define an alternative way of demonstrating this phenomenon on CMR that will provide additive diagnostic power due to better spatial resolution.

## Methods

64 retrospective patients were included in the study. 23 of these had CMR and surgical diagnosis of constrictive pericarditis. Of the remaining 41 patients, 19 were diagnosed as normal and 22 had alternative diagnoses of non-ischaemic cardiomyopathies (Restrictive cardiomypathy, hypertrophic cardiomyopathy, ARCV, non.compaction, Fabry's disease and dilated cardiomyopathy). We identified annular excursion by longitudinal motion of the medial and lateral mitral annulus in a 4 chamber SSFP view perpendicular to the transverse plane. Annular excursion was measured in end diastole and end systole. Annular excursion differences were calculated by subtracting the lateral annular excursion from that of the medial during the diastole and systole. A negative value indicated paradoxical motion. We excluded studies with known wall motion abnormalities, severe valvular heart diease, prosthetic heart valves and poor image resolution due artifacts.

## Results

18 (78%) of the patients with constrictive disease had paradoxical excursion of the mitral valve annulus. Mean annular excursion difference was *-0.88* (*-5.6 to 3.57*).

Of those without constrictive disease 37 (90%) did not show annulus paradoxus. This included 84% of the normal patients and 95% of those with other diagnoses. In this non-constrictive group the mean annular excursion difference was *2.31* (*-1.9 to 6.37*)(p<0.05 compared to constrictive group). *Normal group: 2.41*(*-1.93 to 5.61*)*. Other diagnosis group: 2.45* (*-0.82 to 6.37*)*.*

Using negative annular excursion differences on MRI (annulus paradoxus) for constrictive cardiac disease our results showed a sensitivity of 78%, specificity of 90%, positive predictive value of 82% and negative predictive value of 88%.

## Conclusions

This study shows that annulus paradoxus is a specific and highly reproducible marker of constrictive pericarditis that can be accuratly estimated from 4 chamber SSFP cine CMR images. Unlike echocardiography, we are not dependent on body habitus and opertor skill to obtain high quality images and perform doppler interrogation.

## Funding

None.

**Figure 1:**
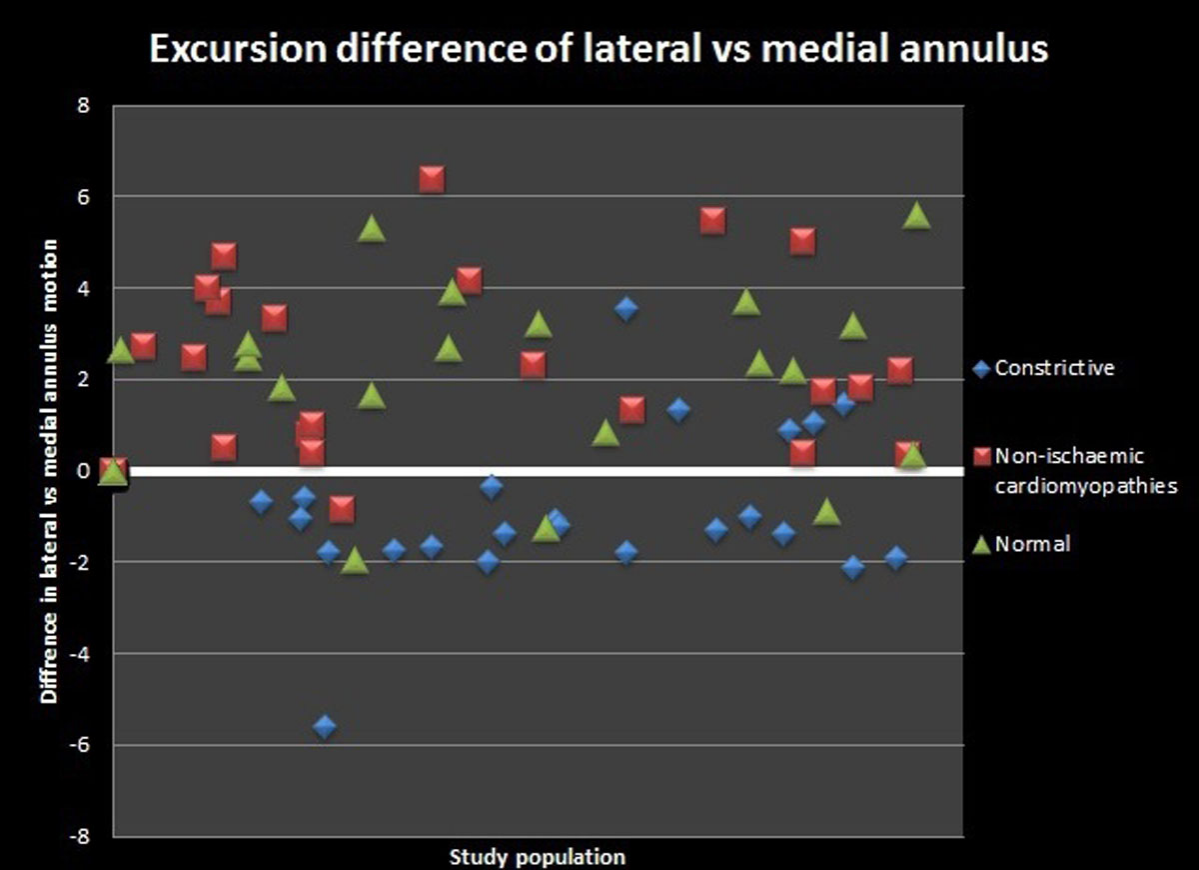
Scatter plot of excursion differences in the three groups

**Table 1:** Mean annular motion and differences.

	Number	Mean medial annular Motion (mm)	Mean lateral annular Motion (mm)	Mean difference (negative indicating Paradoxical motion)
Study Population	64	8.80	9.96	1.16

Pericardial constriction	23	9.05	8.16	-0.88

Non Pericardial constriction	41	8.66	10.97	2.31

Non ischemic cardiomyopathies	22	6.31	8.77	2.46

Normal	19	11.37	13.52	2.14

P-values: Pericardial constriction vs.				

Non Pericardial constriction		0.63	<0.0014	<0.0005

Non-ischaemic cardiomyopathies		0.0028	0.50	<0.0005

Normal		0.003	<0.0005	<0.0005

